# A randomized cross-over study of inhalation of diesel exhaust, hematological indices, and endothelial markers in humans

**DOI:** 10.1186/1743-8977-10-7

**Published:** 2013-03-26

**Authors:** Ranjini M Krishnan, Jeffrey H Sullivan, Chris Carlsten, Hui-Wen Wilkerson, Richard P Beyer, Theo Bammler, Fred Farin, Alon Peretz, Joel D Kaufman

**Affiliations:** 1 Departments of Medicine, University of Washington, Seattle, WA, USA; 2 Environmental and Occupational Health Sciences, 3 Epidemiology University of Washington, Seattle, WA, USA; 3 Epidemiology, School of Medicine and School of Public Health, University of Washington, Seattle, WA, USA

## Abstract

**Background:**

Exposure to traffic-related air pollution (TRAP) is considered a trigger for acute cardiovascular events. Diesel Exhaust (DE) is a major contributor to TRAP in the world. We evaluated the effect of DE inhalation on circulating blood cell populations, hematological indices, and systemic inflammatory cytokines in humans using a specialized facility.

**Methods:**

In a randomized double-blind crossover study balanced to order, 17 metabolic syndrome (MetS) and 15 healthy subjects inhaled filtered air (FA) or DE exposure in two-hour sessions on different days with a minimum 2-week washout period. We collected blood pre-exposure, 7, and 22 hours after exposure initiation and measured the complete blood count and differential. We performed multiplex cytokine assay to measure the changes in the systemic inflammatory cytokines, and endothelial adhesion molecules (n=15). A paired analysis compared the effect of DE and FA exposures for the change from pre-exposure to the subsequent time points.

**Results:**

A significant increase in the hematocrit was noted 7 hrs after DE [1.4% (95% CI: 0.9 to 1.9%)] compared to FA exposure [0.5% (95% CI: -0.09 to 1.0%); p=0.008. The hemoglobin levels increased non-significantly at 7 hrs post DE [0.3 gm/dL (95% CI: 0.2 to 0.5 gm/dL)] versus FA exposure [0.2 gm/dL (95% CI: 0 to 0.3 gm/dL)]; p=0.06. Furthermore, the platelet count increased 22 hrs after DE exposure in healthy, but not in MetS subjects [DE: 16.6 (95% CI: 10.2 to 23) thousand platelets/mL versus [FA: 3.4 (95% CI: -9.5 to 16.3) thousand platelets/mL)]; p=0.04. No DE effect was observed for WBC, neutrophils, lymphocytes or erythrocytes. Using the multiplex assay, small borderline significant increases in matrix metalloproteinase-9, interleukins (IL)-1beta, 6 and 10 occurred 7 hrs post exposure initiation, whereas E-selectin, intercellular adhesion molecule-1, and vascular cell adhesion molecule -1, and myeloperoxidase 22 hrs post exposure.

**Conclusions:**

Our results suggest that short-term DE exposure results in hemoconcentration and thrombocytosis, which are important determinants of acute cardiovascular events. Multiplex assay showed a non-significant increase in IL-1β and IL-6 immediately post exposure followed by myeloperoxidase and endothelial activation molecules. Further specific assays in a larger population will improve our understanding of the systemic inflammatory mechanisms following acute exposure to TRAP.

**Clinical trials registration number:**

Study was conducted between 2004 to 2006, prior to expectation for registration.

## Background

Exposure to traffic related air pollution has been shown to trigger acute cardiovascular events and deaths primarily due to myocardial infarction [1-4]. Although air pollution consists of a heterogeneous mixture of gaseous and particulate matter, adverse cardiovascular events are most strongly associated with exposure to fine particulate matter (PM_2.5_), especially traffic sources of which diesel exhaust (DE) is a principal source [5,6]. DE particles readily deposit within human alveoli and may contribute to the biological toxicity eliciting systemic inflammation and altered coagulability, or both. Air pollutant components may induce these responses through vascular endothelial cells, leukocytes, and/or platelets, with expression of inflammatory cytokines, cellular adhesion molecules, viscosity of blood, and coagulation factors [7]. Activation of these inflammatory pathways then potentially lead to increased vascular reactivity or vasoconstriction, [8,9] endothelial dysfunction, [10] and possibly plaque rupture triggering acute myocardial infarction or ischemia.

Prior panel studies and experimental studies have shown that traffic related air pollution is associated with increased systemic inflammatory cytokines [11]. Evidence from in-vitro studies demonstrated that particulate matter-exposed alveolar macrophages induce cytokine expression [12] and experimental studies assessed the bone marrow stimulatory response in terms of cell counts [13]. We and other groups have used controlled DE exposure studies to understand the mechanisms of the effects of short-term exposure to DE on biological pathways in humans.. We previously showed that there was no effect of DE on markers of coagulation in healthy subjects and in metabolic syndrome subjects [14,15]. It is possible that DE exposure can activate systemic inflammation in human subjects independent of activating the coagulation cascade. Therefore, we hypothesized that DE would affect peripheral blood cell counts, hematological indices, and systemic cytokine production in healthy and metabolic syndrome subjects.

## Results

### Baseline characteristics of subjects

Demographic information for the study participants is shown in Table 1. Fifteen healthy subjects and seventeen metabolic subjects were enrolled in the study. Of these, thirteen subjects (five with metabolic syndrome) had complete information for the multiplex assay. Fifteen healthy subjects and 17 with metabolic syndrome had complete blood counts with differential. The mean age for the healthy subjects was 28 and that of the metabolic subjects was 40. Levels of gaseous co pollutants were low, with nitrogen dioxide ranging from 15–30 ppb and carbon monoxide ranging between 0.2 to 0.7 ppm as reported earlier [14].

**Table 1:** Demographic information of the study participants

**Baseline characteristics**	**Healthy subjects (n=15)**	**Metabolic subjects (n=17)**
**Age, (years)**	28 ± 8	40 ± 8
**Gender, M:F**	11:2	8:6
**Race (Caucasian: other)**	10:3	8:3
**BMI (kg/m**^**2**^**)**	24.7 ± 1.5	40.1 ± 8.1
**SBP (mm Hg)**	115 ± 9	120 ± 9
**DBP (mm Hg)**	71 ± 10	81 ± 7
**Glucose (mg/dL)**	89 ± 5	99.5 ± 9
**Total Cholesterol (mg/dL)**	151 ± 30	193 ± 26
**LDL (mg/dL)**	88 ± 28	121 ± 22
**HDL (mg/dL)**	49 ± 12	37 ± 5
**TGL (mg/dL)**	74 ± 43	189 ± 127
**Platelets (cells/mm**^**3**^**)**	220 ± 43	223 ± 51
**Total WBC (cells/mm**^**3**^**)**	5.07 ± 1.36	5.48 ± 0.71

All of the values reported are mean ± SD unless otherwise noted. Differences between healthy and metabolic syndrome subjects were statistically significant, with the exception of race and sex (M-Male; F-Female). SBP indicates systolic blood pressure; DBP, diastolic blood pressure; LDL, low-density lipoprotein; HDL, high-density lipoprotein. DBP and triglyceride *P*≤0.01; all others *P*≤0.001.

### Systemic effects of diesel exhaust on the hematological indices in the peripheral blood

We did not find any significant changes in the total white blood cell count, neutrophils, or lymphocytes between the exposures. Seven hours following exposure onset, participants tended to have higher hematocrits, likely due to hemoconcentration, regardless of exposure situation. However, the increase in the hematocrit 7-hours post DE exposure was higher than with FA exposure [*Mean DE effect was 1.0% (95% CI: 0.3 to 1.6%)*; p=0.008]. This effect was more pronounced in the metabolic syndrome subjects [*Mean DE effect was 1.1% (95% CI: 0.2 to 2.1%*); p=0.02] compared to healthy normal subjects [*Mean DE effect was 0.7% (95% CI: -0.4 to 1.9%)*; p=0.19] at 7 hours post exposure. (Table 2) We also adjusted for perception of the exposure, which did not affect these results. Similar increase was seen for hemoglobin concentration with borderline significance [*Mean DE effect was 0.2 gm/dL (95% CI: -0.01 to 0.4 gm/dL; p=0.06 for hemoglobin)],* but not erythrocyte count. (Table 2) Further, we also stratified our analysis based on the oxidative stress gene; Glutathione S-Transferase M1 (GSTM1). Results indicated that the presence of the GSTM1 wild-type genotype appeared to modify DE effects on hematocrit, but not for the other endpoints particularly platelets [Additional file 1: Table S1].

**Table 2:** Changes in the hematological indices in all subjects exposed to DE and Filtered Air (FA)

**Hematological indices**	**n**	**Change from baseline to 7 h Mean ± SE**		**DE effect Δ baseline - 7 h (95% CI)**	**n**	**Change from baseline to 22 h Mean ± SE**		**DE effect Δ baseline - 22 h (95% CI)**
		**Filtered air**	**DE-200**			**Filtered air**	**DE-200**	
*Leukocytes (x1000 cells/μL)*	26	0.4 ± 0.2	0.4 ± 0.2	-0.0 (-0.3 to 0.3)	24	0.03 ± 0.1	0.1 ± 0.2	0.1 (-0.3 to 0.4)
*Neutrophils (x1000 cells/μL)*	24	0.4 ± 0.1	0.5 ± 0.2	0.0 (-0.4 to 0.4)	24	0.2 ± 0.1	0.2 ± 0.1	0.1 (-0.3 to 0.4)
*Lymphocytes (x1000 cells/μL)*	25	0.03 ± 0.1	-0.01 ± 0.1	-0.04 (-0.2 to 0.1)	23	-0.1 ± 0.05	-0.1 ± 0.04	-0.03 (-0.2 to 0.1)
*Monocytes (x1000 cells/μL)*	24	0.01 ± 0.02	-0.01 ± 0.03	-0.02 (-0.1 to 0.1)	21	-0.03 ± 0.02	-0.002 ± 0.04	0.03 (-0.1 to 0.1)
*Erythrocytes (million cells/μL)*	30	0.1 ± 0.02	-0.2 ± 0.2	-0.2 (-0.7 to 0.2)	28	0.1 ± 0.03	0.2 ± 0.3	0.1 (-0.5 to 0.7)
*Platelets (x1000 cells/μL)*	24	9.2 ± 3.4	11.4 ± 2.8	2.2 (-4.2 to 8.7)	22	4.5 ± 2.9	10.1± 2.6	5.5 (-2.8 to 13.8)
*Hemoglobin (gm/dL)*	24	0.2 ± 0.1	0.3 ± 0.1	0.2 (-0.0 to 0.4)	24	0.3 ± 0.1	0.5 ± 0.1	0.1 (-0.1 to 0.4)
*Hematocrit (%)*	26	0.5 ± 0.3	1.4 ± 0.2	1 (0.3 to 1.6)*	23	1.6 ± 0.4	1.1 ± 0.3	0.5 (-0.5 to 1.4)

Data are shown for the absolute mean differences from pre-exposure to 7 hours (Δ Baseline-7 h) and 22 hours (Δ Baseline-22 h) of exposure initiation. DE effect refers to the effect attributable to DE calculated as [(Post –pre DE exposure - (Post-pre) FA exposure] displayed as the absolute change in each of the indices.

*Indicates a P-value of 0.008; SE represents standard error; CI – Confidence Interval.

### Inhalation of diesel exhaust may increase platelet count in healthy subjects

**Figure 1:**
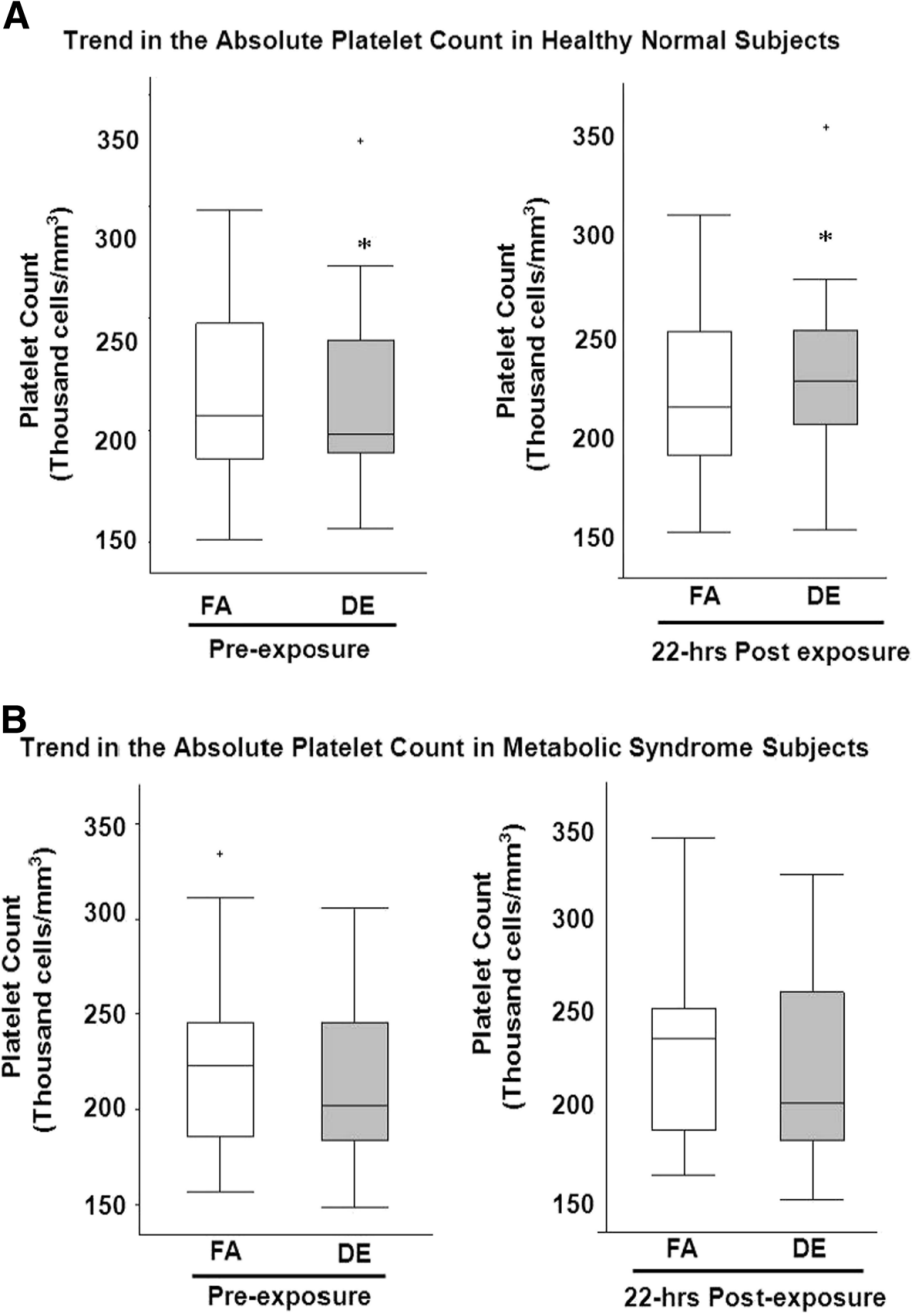
**Absolute Platelet Count in Healthy Normal Subjects (A) and Metabolic Syndrome Subjects (B) following exposure. **Figure showing the distribution of the platelet count at the different time points in (**A**) Healthy subjects and (**B**) Metabolic Syndrome subjects. Each panel depicts medians (bars) and interquartile ranges (whiskers) for platelet count associated with each exposure at baseline (pre-exposure) and 22 hours post exposure initiation. Crosses above the panels represent those that are outside of the interquartile range; * indicates P=0.04 for the difference between DE pre-exposure and 22hours post exposure in healthy normal subjects. FA=filtered air; DE=200 μg/m^3 ^(PM_2.5_) DE.

The peripheral blood platelet count progressively increased at 7 and 22 hours following DE exposure among all the participants [Mean DE effect at 7 hours was 2.2 (95% CI: -4.2 to 8.6) thousand platelets/mL) and at 22 hours was 5.5 thousand platelets/mL (95% CI: -2.8 to 13.8 thousand platelets/mL)] [Table 2]. The finding appeared larger in the healthy subjects, for whom the increase in the platelet count at 22 hours following DE exposure averaged 16.6 (95% CI: 10 to 23) thousand platelets/mL) compared to filtered air (FA) [3.4 (95% CI: -9.5 to 16.3) thousand platelets/mL)]. The mean DE effect in healthy subjects was 13.2 (95% CI: 0.7 to 25.6) thousand platelets/mL; p=0.04 increase at 22 hours. In contrast, the platelet count did not increase 22 hours after exposure in the metabolic syndrome subjects (Mean DE effect was (Mean DE effect was 0.9 (95% CI: -10.5 to 12.3) thousand platelets/mL; p=0.9) [Figure 1].

### Effects of diesel exhaust on endothelial adhesion molecules and inflammatory cytokines: results from the multiplex cytokine assay

Using the multiplex assay, we found that the endothelial cell adhesion molecules E- selectin, intercellular adhesion molecule-1 (ICAM-1), and vascular cell adhesion molecule-1 (VCAM-1) showed a small trend toward increase in the plasma following DE exposure when compared to FA exposure at 22 hours, but variability in measurements was very high and no significant findings were detected (Table 3). Changes related to DE in multiplex measures of cytokines and inflammatory biomarkers were not statistically significant but suggested interesting trends. Myeloperoxidase increased slightly with DE at both time points, while MMP-9 was only elevated at 7 hours after exposure and adiponectin only at the 22-hour time point. Among the inflammatory cytokines, interleukin-1beta (IL-1β) and IL-10 increased at 7 and 22 hours post DE exposure initiation. IL-6 increased 1.1 fold immediately after exposure followed by a decline 24 hours later (Table 4).

**Table 3:** Endothelial activation markers measured by the multiplex assay in subjects exposed to DE and FA

**Endothelial biomarkers**	**N**	**Δ Baseline - 7 h Fold change ± SE (pg/dl)**		**DE Effect (Δ Baseline - 7 h) [fold change] (95% confidence interval)**	**N**	**Δ Baseline - 22 h Fold change ± SE (pg/dl)**		**DE Effect (Δ Baseline - 22 h) [fold change] (95% confidence interval)**
		**Filtered air**	**DE-200**	**P-values**		**Filtered air**	**DE-200**	**P-values**
E-selectin	15	0.0 ± 0.1	0.02 ± 0.1	0.02 (95% CI: -0.3 to 0.3)	15	0.0 ± 0.1	0.1 ± 0.1	0.1 (95% CI: -0.2 to 0.4)
				P=0.8				P=0.4
ICAM-1	15	0.0 ± 0.1	0.1 ± 0.1	0.2 (95% CI: -0.3 to 0.6)	17	0.04 ± 0.1	0.1 ± 0.1	0.1 (95% CI: -0.3 to 0.5)
				P=0.5				P=0.6
VCAM-1	15	0.02 ± 0.1	0.1 ± 0.1	0.03 (95% CI: -0.2 to 0.3)	17	-0.1± 0.1	0.1± 0.1	0.2 (95% CI: -0.1 to 0.4)
				P=0.8				P=0.2

Data are shown for the mean log_2 _differences from pre-exposure to 7 hours (Δ Baseline-7 h) and 22 hours (Δ Baseline-22 h) of exposure initiation. SE represents standard error. DE effect refers to the effect attributable to DE calculated as [(Post –pre DE exposure - (Post-pre) FA exposure] for the log_2 _transformed values to obtain fold changes. Positive difference indicates that change in a given marker was relatively more positive over DE-exposure interval, as compared to change over FA exposure interval.

**Table 4:** Inflammatory cytokines measured by the multiplex assay in humans exposed to DE and FA

**Inflammatory cytokines**	**N**	**Δ Baseline -7 h [fold change ± SE] (pg/dL)**		**DE Effect Δ Baseline-7 h [fold change] (95% confidence interval)**	**N**	**Δ Baseline -22 h [fold change ± SE] (pg/dL)**		**DE Effect Δ Baseline-22 h [fold change] (95% confidence interval)**
		**Filtered air**	**DE-200**			**Filtered air**	**DE-200**	
				**P-values**				**P-values**
Myeloperoxidase	16	0.2 ± 0.2	0.4 ± 0.3	0.2	16	0.01 ± 0.2	0.4 ± 0.2	0.4
				(95% CI: -1.1 to 0.7)				(95% CI: -0.2 to 0.9)
				P=0.7				P=0.2
Adiponectin	16	0.1 ± 0.2	-0.02 ± 0.1	0.1	15	-0.1 ± 0.2	0.2 ± 0.1	0.3
				(95% CI: -0.4 to 0.6)				(95% CI: -0.3 to 0.8)
				P=0.6				P=0.3
MMP-9	14	0.0 ± 0.1	0.3 ± 0.2	0.3	15	0.01 ± 0.1	-0.1 ± 0.1	-0.1
				(95% CI: -0.2 to 0.8)				(95% CI: -0.6 to 0.3)
				P=0.2				P=0.5
MCP-1	15	-0.2 ± 0.1	-0.2 ± 0.1	-0.01	15	-0.2 ± 0.1	-0.2 ± 0.1	-0.01
				(95% CI: -0.3 to 0.2)				(95% CI: -0.2 to 0.2)
				P=0.9				P=0.9
VEGF	15	0.1 ± 0.1	0.1 ± 0.1	0.02	16	0.1 ± 0.1	0.1 ± 0.1	0.01
				(95% CI: -0.2 to 0.2)				(95% CI: -0.1 to 0.1)
				P=0.6				P=0.7
Pro-BNP	5	0.3 ± 0.2	-0.2 ± 0.3	-0.4	6	0.1 ± 0.3	-0.1 ± 0.3	-0.2
				(95% CI: -1.5 to 0.7)				(95% CI: -1.0 to 0.6)
				P=0.9				P=0.1
TNF-α	15	-0.1 ± 0.04	-0.1 ± 0.1	0.02	15	0.0 ± 0.1	-0.03 ± 0.1	-0.03
				(95% CI: -1.4 to 0.2)				(95% CI: -0.2 to 0.1)
				P=0.8				P=0.5
IL-1β	9	-0.3 ± 0.2	0.1 ± 0.1	0.5	11	-0.2 ± 0.3	0.1 ± 0.3	0.3
				(95% CI: -0.01 to 1)				(95% CI: -1.1 to 2 )
				P=0.06				P=0.9
IL-6	11	-0.04 ± 0.1	1.1 ± 0.7	1.1	8	0.2 ± 0.1	-0.1 ± 0.1	-0.3
				(95% CI: -0.7 to 3)				(95% CI: -0.6 to 0.1)
				P=0.6				P=0.1
IL-8	15	-0.3 ± 0.1	-0.2 ± 0.2	0.1	16	-0.2 ± 0.1	-0.3 ± 0.2	-0.1
				(95% CI: -0.3 to 0.6)				(95% CI: -0.5 to 0.3)
				P=0.8				P=0.9
IL-10	15	-0.3 ± 0.1	-0.01 ± 0.1	0.3	16	-0.1 ± 0.1	0.1 ± 0.1	0.2
				(95% CI: -0.1 to 0.6)				(95% CI: -0.3 to 0.6)
				P=0.2				P=0.5

Data are shown for the mean log_2 _differences from pre-exposure to 7 hours (Δ Baseline-7 h) and 22 hours (Δ Baseline-22 h) of exposure initiation. DE effect refers to the effect attributable to DE calculated as (Post –pre DE exposure)-(Post-pre FA exposure) for the log_2 _transformed values to obtain fold changes. Positive difference indicates that the change in given marker was relatively more positive over DE-exposure interval, as compared to change over FA exposure interval. SE - Standard Error; CI - Confidence Interval.

Due to concern regarding the imprecision of the multiplex assay, we repeated IL-6 measurements in ten of these participants using a widely used specific ELISA system. We found that the mean change in the IL-6 level following DE exposure was increased at 7 hours following DE [0.6 pg/mL, (95% CI: -0.1 to 1.3 pg/mL)]; but not following FA [0.1 pg/mL, (95% CI: -0.3 to 0.4 pg/mL)] at 7 hours post-exposure.

## Discussion

In this randomized crossover study, we found that a two-hour exposure to diesel exhaust in a controlled exposure facility resulted in a significant increase in the hematocrit immediately post-exposure without an increase in erythrocyte number. This increase appeared more pronounced in the metabolic syndrome subjects. Healthy, but not metabolic syndrome subjects demonstrated an increase in platelet count a day following DE exposure, without any change in other measured circulating blood cell fractions. Using a multiplex assay, we observed small and not significant increases in several proteins with DE compared to FA, including circulating endothelial adhesion molecules, myeloperoxidase, adiponectin, interleukins-1β, 6, and 10. While the finding would not be robust to multiple testing corrections, the increase in IL-1β immediately post-exposure achieved borderline statistical significance in standard assessment.

This is the first experimental study to examine the hematological indices, circulating peripheral cell count and a variety of inflammatory mediators in a chronological manner in humans following DE exposure. DE is a major contributor to traffic related pollution and contains higher fine particulate content. Therefore, our findings provide some support to the hypothesis that acute exposure to traffic related air pollution initiates hemoconcentration, systemic inflammatory process activating the endothelial–blood cell interface, and increases circulating platelets. All these biological processes are potential important steps in mediating adverse cardiovascular events associated with fine particulate matter exposure [5].

Our finding of a rapid increase in hematocrit without any changes in erythrocytes is in contrast to Seaton’s study that first showed a negative association between 3-day average personal PM_10_ exposures and blood parameters such as hemoglobin, packed red cell volume [16,17]. This striking difference could be due to the different specific concentrations and the experimental setting that we employed to study the acute effects of traffic related air pollutants. Other experimental studies have reported peripheral blood counts, but not hematocrit or hemoglobin from normal healthy subjects. Those studies have also examined the systemic markers in humans for only up to 6hrs [18,19]. It is unclear how fine particulate matter exposure would lead to hemoconcentration, but we speculate that this could be due to a combination of mechanisms such as volume status or stress [20-22] that could affect the blood viscosity. It is unlikely that our results could be influenced purely by our subjects’ fluid intake, as they go through much of the study in a fasting state, identical in each exposure day (DE or FA), and blinded to exposure status, and analyses compare the same time of day for each exposure. Since only DE exposure resulted in a significant hemoconcentration, it implies a direct effect on hemoconcentration, though it is also possible that DE exposure was associated with less desire to consume fluids.

Our recent observation of a 5mm Hg increase in the systolic blood pressure immediately after exposure to DE also indicates sympathetic nervous system mediated effects of PM_2.5_ and future studies will address these questions more specifically [23].

Few studies have addressed the direct effect of PM_2.5_ and other ambient air pollutants on platelets. In our study, we found that inhalation of DE in healthy subjects increased platelet count 22 hours following exposure. It is of note that we did not observe an increase in platelets in the metabolic subjects, who were also older and may have an underlying inflammatory process or impaired bone marrow function which could blunt the response seen in the normal healthy subjects (Figure 1). We previously reported little effect of DE on markers of thrombosis including D-Dimer, von-Willebrand factor in metabolic syndrome subjects, [14] or in healthy subjects [15]. In contrast, another group has shown that exposure to DE results in impaired fibrinolysis and recently showed that DE inhalation increases *ex vivo* thrombus formation and *in vivo* platelet activation in healthy subjects [24]. Our observations could be seen as at odds with these prior findings, but might also be due to differences in exposure characteristics or differences in the molecular targets of our outcome measures. Similar to the human experimental studies, animal studies showed that mice exposed to concentrated ambient particles had a significant increase in their platelet count with evidence of platelet activation [25]. Both arterial and venous thrombosis were noted with increased platelet aggregation in hamsters treated with intra-tracheal instillation of DE particles [26]. Although, we have only seen an increase in the platelet count, this is most likely the first process for thrombogenesis, or could be due to a direct bone marrow stimulatory effect as demonstrated in other studies [12] or a reactive thrombocytosis from a systemic inflammatory response.

In the multiplex luminex assay, the trend toward an increase in the endothelial adhesion molecules (E-selectin, ICAM-1, VCAM-1) and inflammatory cytokines (such as interleukin 1β, interleukin-6, interleukin-10, myeloperoxidase, and matrix metalloproteinase-9) are fairly consistent with other air pollution studies; none of these findings were statistically significant and none were robust to adjustment for multiple comparisons. Epidemiological study from children in the Mexico City observed significant increases in inflammatory mediators and vasoconstrictors, including tumor necrosis factor (TNF) alpha, prostaglandin, C-reactive protein, interleukin-1β, and endothelin-1, and down regulation of endothelial adhesion molecules compared to the children from a less polluted city [27]. An *in vitro* experiment using DE particles caused an inflammatory response in macrophages characterized by increased IL-8 and TNF-α concentration. This inflammatory condition generated by macrophages in response to DEP was a potent activator of endothelial cells than direct particle treatment [28,29].

All the results noted in our current study provide some insights into the mechanisms underlying the short-term exposure to air pollution and cardiovascular diseases. While prior experimental studies in animals and humans have shown that exposure to fine particulate matter results in atherosclerosis progression [30-32]. Less is known about the association between particulate matter exposure and atherothrombosis. Atherothrombosis occurs due to interplay between thrombosis, procoagulation, endothelial erosion, and possibly increased viscosity, typically occluding the lumen acutely leading to acute events such as unstable angina, myocardial infarctions [33-35]. We also noted an increase in markers of neutrophil activation such as myeloperoxidase and matrix metalloproteinase-9, but not increased neutrophil count suggesting that DE exposure could promote systemic inflammation by activating neutrophils than by increasing their production. These mechanisms are proposed based on previous epidemiological studies that showed an association between short-term increases in traffic related air pollution exposure and increased incidence of myocardial infarction [1,35] and deep venous thrombosis [36]. While there are not many human experimental studies that have shown an association between air pollution exposure and increased hematocrit, prior cardiovascular epidemiology studies have shown an association between elevated hematocrit and coronary artery disease suggesting that elevated hematocrit is associated with a greater risk of acute coronary syndromes, which is hypothesized to be from increased whole blood viscosity [37-39]. Similar to this, a panel study of elderly patients with CAD showed an increase in inflammatory cytokines (interleukin-6), oxidative biomarkers (myeloperoxidase), and platelet activation [11,40].

Endothelial adhesion molecules have been considered as markers of endothelial injury, [41] and have been associated with adverse cardiovascular events [42]. Endothelial activation results in up regulation of leukocyte adhesion proteins, attracts monocytes through release of growth factors and pro-inflammatory cytokines, and initiates vascular inflammation [43]. However, *in vivo* intra-vital microscopy studies have shown that platelet-endothelium adhesion can occur in intact endothelium without activation of the coagulation cascade [44] through activation of platelet and endothelial adhesion molecules like ICAM-1, VCAM-1, and E-selectin. Our previous study did not show a significant increase in the pro-coagulant and pro-thrombotic factors including von Willebrand factor, plasminogen activator inhibitor-1, and D-dimer, suggesting that the activation of the coagulation cascade may not occur as an early signal in our experimental model or could be due to lack of adequate sensitivity of these assays [14,15]. Based on our observations, we hypothesize that platelet-endothelial activation rather than procoagulation could be one of the underlying mechanisms in subjects exposed to DE that have to be confirmed in future studies.

The major strengths of our study are the use of a rigorously controlled experimental design in which each subject serves as their own control, the enrollment of both healthy subjects and those with metabolic syndrome, and observations at early (7 hours following exposure initiation) and late (22 hours) time points. Using a randomized crossover study design, we were also able to study the effect of DE directly on a broad range of cytokines and cell counts in a sequential manner.

Our study has several limitations. We would expect an experimental study like ours to have more robust abilities to detect an effect, if present, than observational designs, but our findings are not strongly positive. Several factors may explain this. Although we were able to assess the effect on multiple cytokines using the most sensitive assay, [45] there was wide variability in the calculated concentrations. Prior studies have validated the multiplex assay in comparison to the ELISA, using samples other than plasma [46,47]. Other investigators who employed this assay in inflammation research [48] and in non-diseased healthy subjects, [49] reported that their findings are difficult to interpret due to the low sensitivity of this assay from multiple interactions with other plasma proteins and poor threshold of detection for the inflammatory markers such as IL-1β, TNF-α and IL-6. In addition to these, our study samples utilized for the multiplex assay also came from a subset of heterogeneous population consisting of normal healthy subjects and metabolic syndrome. Furthermore, our experimental findings cannot distinguish if the source of the inflammatory markers is pulmonary or systemic in origin. It is also unclear from our study if the increased platelet in the circulation is from bone marrow stimulation or a reactive thrombocytosis phenomenon due to increased inflammation. Hence, to address these issues, we plan to use more sensitive, specific ELISA assays for the endothelial adhesion molecules, neutrophil inflammatory markers, cytokines, and platelet activation assays in our future studies. Clinical studies such as these are logistically difficult, and the small number of participants seems an obstacle to obtaining sufficient statistical power. Nevertheless, we were well powered to detect clinically significant changes in our outcome parameters.

## Conclusions

In a well-controlled experimental setting using DE as a model traffic-related air pollutant, our results suggest that acute exposure to DE might result in an increase in endothelial adhesion molecules, hemoconcentration, and systemic inflammatory cytokines such as interleukins 1β, 6, and 10, as early as 7 hours post-exposure initiation, followed by thrombocytosis 22 hours after exposure initiation. Taken together, these findings suggest that platelet-endothelial activation may be an early pathway for the evolution of vascular phenomena by which traffic-related air pollutants trigger myocardial infarction and ischemic stroke.

## Methods

### Subject recruitment and inclusion criteria

Subjects were eligible to participate in the study if they fulfilled the following inclusion criteria: age 18–49 years; non-smoking status at least 6 months prior to recruitment; no history of on-going medical care for heart disease or asthma; lack of arrhythmia or ischemia on electrocardiograph (ECG); normal spirometry (MicroDL, Micro Medical Ltd, Kent, UK); and having the metabolic syndrome (3 or more of the following 5 criteria) [50] elevated waist circumference [men, equal to or greater than 40 inches; women, equal to or greater than 35 inches]; elevated triglycerides [equal to or greater than 150 mg/dl]; reduced HDL cholesterol [men, less than 40 mg/dl; women, less than 50 mg/dl]; elevated blood pressure [equal to or greater than 130/85 mm Hg]; elevated fasting glucose [equal to or greater than 100 mg/dl]). Subjects were required to be off blood thinning medications, statins, and antioxidants. Women of childbearing age underwent a urine pregnancy test at screening and before each exposure, and were instructed to practice effective contraception during the study. All subjects gave a written informed consent prior to the screening process. The consent form and study protocol were approved by the University of Washington Human Subjects Review Division.

### Study design

Using a randomized double-blinded crossover study, 17 subjects with metabolic syndrome and 15 healthy subjects were exposed to FA or DE (at 200 μg PM_2.5_/m^3^) in two-hour sessions on different days after a 2-week washout period. DE exposure was calibrated based on the mass of particles less than 2.5 microns in diameter (PM_2.5_), and measured continuously using a tapered element oscillating microbalance. (TEOM 1400A PM_2.5_, Rupprecht & Patashnick Co., Albany, NY) Exhaust was generated from a 2002 turbocharged direct injection 5.9-L Cummins B series engine (6BT5.9G6, Cummins, Inc., Columbus, IN) via 100-kW generator distributed to a 116-m^3^ exposure room after a two-phase dilution of exhaust provided a total dilution of 400:1.

Temperature and relative humidity were maintained at 18°C and 60%, respectively for both filtered air and DE exposures*.* Particle source composition (carbon and trace elements) was similar to that of the U.S. Environmental Protection Agency (EPA) light-duty diesel profile. Based on multistage impactor-collected samples, the facility's DE particle mass median diameter was 0.10 μm (σ_g_ = 1.15) [51]*.* Particle count per cubic centimeter for our exposure scenario was 2.8×10^3^ for FA and 5.3×10^4^ for DE exposures [23].

Subjects fasted overnight and during the exposure. They ate a standardized meal, with the same food content and quantity, after each exposure. Exposure began consistently within 30 min of 9 a.m. on weekdays and lasted 2 hours, during which time subjects were resting. We drew blood samples two hours prior to exposure initiation, 7 and 22 hours post exposure initiation. All researchers, nurses and technicians participating in the study were blinded to exposure type, with the exception of the exposure engineer. To evaluate blinding adequacy, subjects were asked during exposure to estimate the level of DE in the chamber. We considered this perception of exposure in our analysis and used as a categorical variable to adjust for the blood count analyses. All subjects gave written informed consent. The University of Washington Human Subjects Division approved the consent form and study protocol.

### Multiplex bead-based cytokine assays

Plasma samples were analyzed using the Lincoplex Human Cardiovascular Disease Panel 1 (CVD1) and Panel 3 (CVD3) (currently called the MILLIPLEX MAP Human Cardiovascular Disease Panel) and the Human Custom Immunoassay 3-plex kit (Millipore, Billerica, MA) according to the manufacturer’s instructions. The CVD1 was used to measure cytokines soluble E-selectin, soluble VCAM-1, soluble ICAM-1, MMP-9, Myeloperoxidase (MPO), and Adiponectin whereas CVD3 was to measure cytokines IL-1, IL-6, IL-8, IL-10, TNF-α, MCP-1, NT-proBNP and VEGF. Also, the Human Custom Immunoassay 3-plex was used to measure cytokines level of IL-1β, IL-6, and MCP-1. Briefly, 25 μL of each standard from series of 4-fold dilution and samples were incubated with target capturing beads on a 96-well plate for 16 hours at 4°C, followed by one hour of incubation with specific biotinylated detection antibodies at room temperature. Next, Streptavidin-Phycoerythrin was added to each well and incubated for 30 minutes at room temperature. Filtration and washing were incorporated after each incubation step with wash buffer using vacuum manifold. Samples were re-suspended in sheath fluid prior to reading using the Luminex 100TM (Austin, TX) suspension array reader. Each sample was analyzed in duplicates. We ran this analysis on 13 healthy subjects and 5 metabolic subjects, but the final number of samples for each analyte at the different time points was dependent on the individual limit of detection.

### Human IL-6 assays

Quantitative analysis of IL-6 was performed using the Quantikine High Sensitivity Human IL-6 ELISA kit (R&D, Minneapolis, MN) according to the manufacturer’s instructions. Briefly, 100 μL of each standard from series of 2-fold dilution and samples were incubated on a 96-well microplate for two hours, followed by two hours of incubation with IL-6. Washing were incorporated after each incubation steps. Substrate solution was added to the samples and incubated for one hour, followed by 30 minutes incubation of amplifier solution. Finally, plates were read at 490nm with the Spectra Max Plus (Molecular Devices, Sunnyville, CA) immediately after addition of stop buffer. Each sample was analyzed in duplicates.

### CBC with differential count and genotyping

CBC with differential count was measured in an automated analyzer at the University of Washington Medical Center clinical laboratories, Seattle, WA. We also used the genomic DNA from the frozen buffy coat samples to genotype for the Glutathione S-Methyl Transferase-1 (GSTM1) based on the allele-specific multiplex PCR assay [Additional file 1: Table S1].

### Statistical analysis

Descriptive data are shown as mean ± SD or as percentage. Cytokine analyses were performed following logarithmic transformation to improve normality of data. Paired t-test was performed comparing DE and FA exposures for the change from pre-exposure to 7 hours or 22 hours after exposure initiation. We analyzed the change attributed to DE exposure as [post-DE – pre-DE] – [post-FA – pre-FA]. We also evaluated data for evidence of carryover effects and adjusted for perception.

## Abbreviations

PM2.5: Fine particulate matter; ICAM-1: Intercellular adhesion molecule; VCAM-1: Vascular cellular adhesion molecule; E-selectin: E-selectin; IL-6: Interleukin-6; IL-1β: Interleukin-1beta; TNF- α: Tumor necrosis factor-alpha; MMP-9: Matrix Metalloproteinase-9; MCP-1: Monocyte Chemotactic Protein-1; MPO: Myeloperoxidase; VEGF: Vascular Endothelial Growth Factor.

## Competing interests

The authors declare that they have no competing interests.

## Authors’ contributions

RMK performed data analysis, wrote, and edited the manuscript. JS conceived the study, and supervised design and coordination of data collection. HW carried out the Luminex multiplex assay analyses and the ELISA for IL-6; FMF participated in the design of the experimental assays and provided input on the results. CC provided input for data analysis and editing the manuscript. DB and TB provided statistical consultation on the data analysis. JDK had overall responsibility for the experiments, conceived the study and its design and coordination, and edited the manuscript. All authors read, corrected, and approved the manuscript.

## Supplementary Material

Additional file 1: Table S1 Changes in the Hematocrit in subjects exposed to Diesel Exhaust (DE) and Filtered Air (FA) based on the Glutathione-S-Transferase M1 (GSTM1) status of the participants. P-values are shown based on the paired t-test results from stratified analysis. Interaction testing was not significant. Click here for file
