# Supplementary material for: A randomized cross-over study of inhalation of diesel exhaust, hematological indices, and endothelial markers in humans
Source: Part Fibre Toxicol. 2013 Mar 26;10:7. doi: 10.1186/1743-8977-10-7 (PMC3637197; doi:10.1186/1743-8977-10-7)
Supplement: Additional file 1: Table S1 — Changes in the Hematocrit in subjects exposed to Diesel Exhaust (DE) and Filtered Air (FA) based on the Glutathione-S-Transferase M1 (GSTM1) status of the participants. P-values are shown based on the paired t-test results from stratified analysis. Interaction testing was not significant. [file 1743-8977-10-7-S1.docx]

A randomized cross-over study of inhalation of diesel exhaust, hematological indices, and endothelial markers in humans

Ranjini M Krishnan^1, 2*^
^*^ Corresponding author
Email: ranjik@u.washington.edu

Jeffrey H Sullivan^2^
Email: sullivan.jh@ghc.org

Chris Carlsten^2^
Email: carlsten@mail.ubc.ca

Hui-Wen Wilkerson^2^
Email: jasminew@u.washington.edu

Richard P Beyer^2^
Email: dbeyer@u.washington.edu

Theo Bammler^2^
Email: tbammler@u.washington.edu

Fred Farin^2^
Email: freddy@u.washington.edu

Alon Peretz^2^
Email: alonpz@gmail.com

Joel D Kaufman^1,2,3^
Email: joelk@u.washington.edu

^1^ Departments of Medicine, ^2^Environmental and Occupational Health Sciences, and ^3^Epidemiology, School of Medicine and School of Public Health, University of Washington, Seattle, WA, USA.

**Background:**

The glutathione S-transferase M1 (GSTM1)-null variant is a common copy number variant associated with adverse cardiovascular and pulmonary outcomes, including coronary heart disease and asthma, with evidence of important gene-by-environment interactions with exposures to oxidative stress such as air pollution or cigarette smoking. Prior studies suggest that the null polymorphisms of glutathione S-transferase M1 or T1 (GSTM1/GSTT1) may affect the ability to detoxify or activate chemicals in cigarette smoke and may modify the effect on coronary heart disease. [1]

**Methods:**

We used the buffy coat DNA from the frozen samples to genotype for the Glutathione S Transferase M1 genotype in all our participants using a multiplex PCR based assay as published before. [2, 3]

**Results:**

About 42% of our study population demonstrates the deletion genotype (homozygous null) for GSTM1 (Glutathione S-Transferase M1). In our stratified analysis, we found that the GSTM1 wild type genotype appeared to modify the DE effects on hematocrit significantly (Supplemental Table), but not for the other endpoints particularly platelets. Thus, the effect modification was not in the hypothesized direction, as we observe more effect with the “wild type” genotype than with the null genotype as reported in other studies. [4-6] Future studies on large samples are needed to clarify the role of the GSTM1 genotype on blood indices and systemic inflammatory markers and their interaction with air pollution.

| **Hematological Indices** | **n** | **Change from Baseline to 7h**  **Mean ± SE** | | **DE Effect**  **ΔBaseline - 7h**  **(95% CI)** | **P-values** | **n** | **Change from Baseline to 22h**  **Mean ± SE** | | **DE Effect**  **ΔBaseline - 22h**  **(95% CI)** | **P-values** | |
| --- | --- | --- | --- | --- | --- | --- | --- | --- | --- | --- | --- |
|  |  | **Filtered Air** | **DE-200** |  |  |  | **Filtered Air** | **DE-200** |  |  |  |
| **ALL PARTICIPANTS** | | |  | | | | | | | | |
| *Hematocrit (%)* | 26 | 0.5 ± 0.3 | 1.4 ± 0.2 | 1  (0.3 to 1.6)* | 0.008 | 23 | 1.6 ± 0.4 | 1.1 ± 0.3 | 0.5  (-0.5 to 1.4) | 0.3 | |
| *GSTM1 null* | 10 | 1.1 ± 0.5 | 1.5 ± 0.6 | 0.4  (-1.1 to 1.9) | 0.6 | 7 | 0.6 ± 0.5 | 1.4 ± 0.6 | -0.9  (-2.5 to 0.8) | 0.3 | |
| *GSTM1wild type* | 13 | 0.8 ± 0.3 | 1.5 ± 0.2 | 1.5  (0.8 to 2.1)* | 0.0005 | 13 | 0.8 ± 0.4 | 2.3 ± 0.4 | 1.5  (0.4 to 2.7)* | 0.01 | |
| **HEALTHY NORMALS** | | | | | | | | | | | |
| *Hematocrit (%)* | 11 | 1.0 ± 0.5 | 1.7 ± 0.5 | 0.7  (-0.4 to 1.9) | 0.2 | 12 | 1 ± 0.4 | 1.8 ± 0.5 | -0.8  (-1.9 to 0.4) | | 0.2 |
| *GSTM1 null* | 6 | 1.5 ± 0.8 | 1.7 ± 0.8 | 0.2  (-2.3 to 1.9) | 0.8 | 5 | 0.6 ± 0.7 | 1.8 ± 0.6 | -1.2  (-3.6 to 1.1) | | 0.2 |
| *GSTM1wild type* | 4 | 0.8 ± 0.5 | 1.8 ± 0.3 | 1  (1.7 to 0.2) | 0.1 | 5 | 0.8 ± 0.8 | 2.6 ± 0.9 | 1.8  (-1.2 to 4.8) | | 0.1 |
| **METABOLIC SUBJECTS** | | | | | | | | | | | |
| *Hematocrit (%)* | 15 | 0.1 ± 0.2 | 1.2 ± 0.2 | 1.1  (0.2 to 2.1)* | 0.02 | 15 | 1 ± 0.4 | 1.8 ± 0.4 | 0.8  (-1.9 to 0.4)* | | 0.02 |
| *GSTM1 null* | 7 | 0.4 ± 0.3 | 1.1 ± 0.4 | 1.6  (-0.8 to 2.2) | 0.3 | 5 | 0.6 ± 0.6 | 1 ± 0.5 | 0.4  (-2.8 to 3.6) | | 0.7 |
| *GSTM1wild type* | 7 | -0.4 ± 0.4 | 1.3 ± 0.4 | 1.7  (0.1 to 3.3) | 0.05 | 7 | 2.2 ± 0.6 | 1.2 ± 0.5 | 1  (-0.2 to 2.2) | | 0.1 |

Supplemental Table: Changes in the Hematocrit in subjects exposed to Diesel Exhaust (DE) and Filtered Air (FA) based on the Glutathione-S-Transferase M1 (GSTM1) status of the participants. P-values are shown based on the paired t-test results from stratified analysis. Interaction testing was not significant.

**References:**

1. Li RL, Boerwinkle E, Olshan AF, Chambless LE, Pankow JS, Tyroler HA, Bray M, Pittman GS, Bell DA, Heiss G: **Glutathione S-transferase genotype as a susceptibility factor in smoking-related coronary heart disease.** *Atherosclerosis* 2000, **149:**451-462.

2. Bell DA, Taylor JA, Paulson DF, Robertson CN, Mohler JL, Lucier GW: **Genetic Risk and Carcinogen Exposure - a Common Inherited Defect of the Carcinogen-Metabolism Gene Glutathione-S-Transferase M1 (Gstm1) That Increases Susceptibility to Bladder-Cancer.** *Journal of the National Cancer Institute* 1993, **85:**1159-1164.

3. Chen H, Sandler DP, Taylor JA, Shore DL, Liu E, Bloomfield CD, Bell DA: **Increased risk for myelodysplastic syndromes in individuals with glutathione transferase theta 1 (GSTT1) gene defect.** *Lancet* 1996, **347:**295-297.

4. Saadat M: **Genetic polymorphisms of glutathione S-transferases M1 and T1 modulate hematological changes of individuals chronically exposed to natural sour gas.** *Biochem Biophys Res Commun* 2004, **324:**584-587.

5. Chahine T, Baccarelli A, Litonjua A, Wright RO, Suh H, Gold DR, Sparrow D, Vokonas P, Schwartz J: **Particulate air pollution, oxidative stress genes, and heart rate variability in an elderly cohort.** *Environ Health Perspect* 2007, **115:**1617-1622.

6. Madrigano J, Baccarelli A, Wright R, Suh H, Sparrow D, Vokonas P, Schwartz J: **Air Pollution, Obesity, Genes, and Cellular Adhesion Molecules.** *Occup Environ Med* 2009.
